# Prophylactic and Therapeutic Effects of Interleukin-2 (IL-2)/Anti-IL-2 Complexes in Systemic Lupus Erythematosus-Like Chronic Graft-Versus-Host Disease

**DOI:** 10.3389/fimmu.2018.00656

**Published:** 2018-04-04

**Authors:** Stefan Heiler, Jonas Lötscher, Matthias Kreuzaler, Johanna Rolink, Antonius Rolink

**Affiliations:** Developmental and Molecular Immunology, Department of Biomedicine, University of Basel, Basel, Switzerland

**Keywords:** chronic graft-versus-host-disease, interleukin-2/anti-interleukin-2 complexes, lupus, host regulatory T cells, donor CD8^+^ T cells, interleukin-2 receptor, autoantibodies, immune complex-mediated glomerulonephritis

## Abstract

Murine chronic graft-versus-host-disease (cGvHD) induced by injection of parental lymphocytes into F1 hybrids results in a disease similar to systemic lupus erythematosus. Here, we have used DBA/2 T cell injection into (C57BL/6 × DBA/2)F1 (BDF1) mice as a model system to test the prophylactic and therapeutic effects of interleukin-2 (IL-2)/anti-IL-2 immune complexes on the course of cGvHD. Our findings demonstrate that pretreatment with Treg inducing JES6/IL-2 complexes render BDF1 mice largely resistant to induction of cGvHD, whereas pretreatment with CD8^+^ T cell/NK cell inducing S4B6/IL-2 complexes results in a more severe cGvHD. In contrast, treatment with JES6/IL-2 complexes 4 weeks after induction had no beneficial effect on disease symptoms. However, similar treatment with S4B6/IL-2 complexes led to a significant amelioration of the disease. This therapeutic effect seems to be mediated by donor CD8^+^ T cells. The fact that a much stronger cGvHD is induced in BDF1 mice depleted of donor CD8^+^ T cells strongly supports this conclusion. The contrasting effects of the two different IL-2 complexes are likely due to different mechanisms.

## Introduction

Systemic lupus erythematosus (SLE) is a complex, systemic autoimmune disease affecting multiple organs (1). High titers of autoantibodies binding to nuclear components, including histones and DNA, are characteristic of SLE and are routinely used as a disease marker in clinical diagnosis. Immune complex-mediated glomerulonephritis (ICGN), likely resulting from renal deposition of immune complexes and autoantibodies, is a common and severe clinical manifestation of SLE causing high mortality among affected individuals (2). Although the cellular and molecular events leading to breakdown of tolerance and the emergence of pathologic autoantibodies are still rather obscure, genetic traits clearly play a pivotal role in the susceptibility to SLE (3, 4). Once tolerance is broken either at the T cell or B cell level, self-amplifying/sustaining loops of antigen-presentation and lymphocyte-activation contribute to the generation of high-affinity autoantibodies (5, 6). Notably, the majority of pathogenic autoantibodies found in SLE are somatically hypermutated and class-switched, indicating differentiation and affinity maturation of autoreactive B cells in the germinal centers of secondary lymphoid organs. Moreover, through somatic hypermutation, previously non-autoreactive precursors can also contribute to the pool of self-antigen reactive B cells (7, 8). Follicular helper T (Tfh) cells play important roles in germinal center reactions leading to the generation of high-affinity B cell clones and long-lived memory (9). There is accumulating evidence that aberrant Tfh responses contribute to SLE pathology, and new therapeutic approaches targeting Tfh-associated molecules are currently being tested (10). Up to now, standard SLE therapy depends on general immunosuppressive and anti-inflammatory drugs (11). More recently, the anti-BAFF monoclonal antibody (mAb) belimumab showed beneficial therapeutic effects in combination with standard drugs in clinical studies and has been approved for SLE therapy (12, 13). However, there is still an unmet clinical need for more specific therapies to improve the treatment of lupus.

Autoimmune-prone mice that spontaneously develop lupus-like disease have substantially contributed to a better understanding of genetics underlying disease development through identification of several loci contributing to disease susceptibility (14, 15). Moreover, spontaneously occurring mutations in, or targeted disruption of, specific genes in mice leading to SLE-like symptoms facilitate the identification of molecular events contributing to the pathogenesis of lupus (16).

The chronic graft-versus-host-disease (cGvHD) represents another commonly used mouse model for SLE-like disease and can be induced by transferring CD4^+^ T cells into MHC-II mismatched recipients otherwise not prone to develop SLE-like autoimmunity (17). A well-established strain combination for the induction of cGvHD is the injection of parental DBA/2 (H2^d/d^) lymphocytes into semi-allogeneic (C57BL/6 × DBA/2)F1 (BDF1) (H2^b/d^) recipients (18). These mice develop symptoms closely resembling SLE, including high titers of anti-nuclear antibodies (ANA), anti-isologous erythrocyte (anti-RBC) antibodies, and fatal ICGN (19, 20). The known time point of disease induction facilitates studies on disease kinetics in this model. Moreover, the relatively easiness to manipulate the course of the disease and the rapid kinetics of disease development are, in our opinion, advantages to the spontaneous models mentioned above.

Interleukin-2 (IL-2) is a type I cytokine, produced primarily by conventional T cells, with pleiotropic effects on various cells of the immune system (21). Paradoxically, IL-2 can exert contradictory effects depending on the immunological context. On one hand, IL-2 exerts stimulatory effects on immune responses by expanding effector T cell populations. On the other hand, IL-2 can be immunosuppressive by inducing the proliferation of regulatory T cells (Tregs) that critically depend on IL-2 for homeostasis and to maintain their suppressive capacity (22). Thus, this property makes IL-2 an important regulator of peripheral self-tolerance by balancing the ratio of effector T cells and Tregs. Interestingly, T cells in some SLE patients were shown to be hyperactivated, but at the same time also produce less IL-2 compared to cells from healthy individuals (23, 24). Accordingly, impaired IL-2 production in some SLE patients might account for disturbed Treg homeostasis leading to a breakdown of tolerance to self-antigens.

Initially, IL-2 was discovered and described as growth factor for T cells due to its ability to induce activation and proliferation of T cells *in vitro* (25, 26). Based on these findings, IL-2 was later on used as therapeutic treatment for renal cell carcinoma and metastatic melanoma. A major drawback, however, was the short half-life (~5 min) of IL-2 in the circulation and its toxicity at high doses. Thus, high-dose regimes necessary to achieve a clinical effect were accompanied by severe side effects, including a general vascular leakage syndrome. Additionally, response rates were rather poor at that time (5–10%) (27, 28). This might have in part contributed to the prevailing opinion that IL-2 has only a limited clinical potential. However, already by the 1990s, it was shown that autoimmune symptoms developing in MRL/lpr mice could be efficiently ameliorated by transfection with an IL-2-producing retroviral vector (29). Although this study provided evidence for IL-2 as potential treatment in autoimmune settings, this highly interesting finding was never followed up, likely due to the severe side effects of IL-2 observed in cancer immunotherapy.

More than 10 years ago, Boyman and co-workers elegantly demonstrated that the efficiency of IL-2 treatment could be readily enhanced and at the same time severe side effects could be prevented or largely reduced when IL-2 was administered as an immune complex bound to an anti-IL-2 mAb (30). Moreover, the authors showed that depending on the anti-IL-2 mAb used for the formation of the immune complexes different T cell subsets could be stimulated and expanded. Administration of IL-2 complexes generated with anti-IL-2 mAb JES6.1 (JES6/IL-2) selectively stimulate expansion of Tregs, whereas injection of IL-2 complexes formed by anti-IL-2 mAb S4B6 (S4B6/IL-2) induce predominantly an expansion of the CD8^+^ T cell compartment and to a fewer extend an expansion of NK cells (31).

Structural analysis of IL-2 complexes suggests that mAb JES6.1 blocks epitopes of the IL-2 molecule involved in binding to IL-2 receptor β-chain (IL-2Rβ) and common γ-chain (γc) subunits, thus promoting interaction with IL-2 receptor α-chain (IL-2Rα). This in turn increases the biological availability to cells expressing high-affinity IL-2 receptors (IL-2Rαβγ) like Tregs. In contrast, mAb S4B6 blocks the epitope required for interactions with IL-2Rα, thus favoring interaction with low-affinity IL-2 receptors (IL-2Rβγ) expressed at high levels on CD8^+^ T cells (32, 33). Up to now, the efficiency of IL-2 complexes in immunotherapy has been demonstrated in several murine models. It was shown that JES6/IL-2 complexes promote allograft survival, suppress the development of arthritis, and prevent the induction of experimental autoimmune encephalomyelitis (34–36). In contrast, S4B6/IL-2 complexes have been shown to enhance anti-tumor activity (37, 38). Whether IL-2 complexes might be equally efficient for the treatment of murine SLE-like autoimmune symptoms resulting from cGvHD has not yet been addressed in detail.

In this study, we examined the prophylactic and therapeutic effects of JES6/IL-2 and S4B6/IL-2 complexes on cGvHD. Our findings demonstrate that Treg expansion by JES6/IL-2 complexes, prior to disease induction, protects mice to a large extend from developing cGvHD. On the other hand, therapeutic administration of S4B6/IL-2 complexes 4 weeks after disease induction leads to significant amelioration of the disease. Interestingly, prophylactic treatment with S4B6/IL-2 complexes induces exacerbated cGvHD, whereas treatment of ongoing disease with JES6/IL-2 complexes has no significant effect on disease symptoms. Moreover, we show that donor CD8^+^ T cells are an important factor in cGvHD development. When cGvHD is induced in the absence of donor CD8^+^ T cells, the disease is significantly aggravated, suggesting an inhibitory role of these cells on the course of cGvHD. The potential mechanism by which IL-2 complexes interfere with cGvHD and how donor CD8^+^ T cells contribute to suppression of disease is discussed.

## Materials and Methods

### Mice

(C57BL/6 × DBA/2)F1 (BDF1) and DBA/2 were bred in our animal facility and all mice were maintained under specific pathogen-free conditions. For experiments and analysis, age- and sex-matched mice between 8 and 12 weeks of age were used. Animal experiments were carried out within institutional guidelines (authorization number 1888 and 2434 from Cantonal Veterinarian Office, Basel).

### Preparation of Donor Cells and Induction of GvHD

For preparation of donor cells, spleens and LN (cervical, axillary, brachial, inguinal, and mesenteric) were removed from DBA/2 mice and gently passed through a 40 µm nylon mesh to obtain single cell suspensions in serum-free (SF) IMDM supplemented with 2% FCS (MP Biomedical, USA) and 0.5% Ciproxine (Bayer AG, CH). Spleen cell suspensions were treated with ACK buffer to lyse erythrocytes. Single cell suspensions were pooled, counted by Trypan blue exclusion, and washed in SF IMEM (Sigma-Aldrich, USA) prior to injection. GvHD was induced by i.v. injection of 70 × 10^6^ DBA/2 lymphocytes in a volume of 200 µl SF-IMEM.

### Depletion of Donor CD8^+^ T Cells

In order to obtain donor cell suspensions depleted of CD8^+^ T cells, DBA/2 mice were injected i.v. with 200 µl of 1 mg/ml YTS-156, a rat anti-mouse mAb specific for CD8β, 4 days before the mice were used to prepare cell suspensions. YTS-156 was purified from hybridoma culture supernatant according to standard procedures. The efficiency of CD8^+^ T cell depletion was confirmed by flow cytometry using fluorescent-labeled anti-CD8α-specific mAb (53-6.7).

### Preparation of IL-2 Complexes

For a single injection, 2.5 µg rIL-2 and 7.5 µg anti-IL-2 mAb JES6.1A12 (BioXCell, USA) or S4B6 (purified from hybridoma culture supernatant by standard procedure) were mixed to prepare the IL-2 complexes. After a 30 min incubation at 37°C, the volume was adjusted to 200 µl with sterile PBS and injected i.p. into mice. Control mice were left untreated.

### Detection of Autologous IgG Anti-Erythrocyte Antibodies (Anti-RBC)

For the detection of anti-RBC antibodies in blood of cGvHD mice, Coombs test was performed as described elsewhere (18). Briefly, 100 µl heparinized blood was first diluted in the ratio of 1:20 in PBS containing 2% FCS and 0.1% of 1 M NaN_3_. 25 µl of diluted blood was incubated with 50 µl of 1:200 diluted fluorescein isothiocyanate (FITC)-labeled goat anti-mouse IgG antibody (Jackson ImmunoResearch, USA) for 30 min at 4°C. Cells were washed and bound antibody was detected using a FACSCalibur (BD Bioscience, USA) flow cytometer. Mice were scored positive when the median of fluorescence intensity of staining was increased more than twofold compared to healthy controls.

### Detection of ANA

For the detection of ANA in the sera of cGvHD mice, 8 µm sections of snap-frozen kidneys obtained from RAG2^−/−^ mice were used. Sections were first incubated with 80 µl serum from cGvHD mice diluted from 1:20 to 1:5,120 for 30 min at room temperature (RT) in the dark. After washing, bound ANA were detected by incubation with 80 µl of a 1:200 diluted FITC-labeled goat anti-mouse IgG antibody for 30 min at RT in the dark. The titer was determined by using a fluorescent microscope (Zeiss Axioscope) and was defined as the highest dilution that still gave a specific nuclear staining. Mice containing sera with titers lower than 1:20 were considered as negative for ANA.

### Measurement of Proteinuria

Proteinuria was determined semi quantitatively in weekly intervals using Albustix (Siemens Healthcare Diagnostics Inc., Newark, Delaware). Elevated protein levels in the urine are indicative of failure in kidney function (20). Mice were scored positive when a concentration >3 mg/ml was indicated by color change of the Albustix.

### Immunhistological Analysis

Kidneys from proteinuria positive mice were embedded in OCT-compound (Sakura Finetek, Netherlands) and snap-frozen on dry ice. 8 µm sections were prepared on glass slides, fixed in acetone for 10 min, and dried. For the detection of immune complex deposition in the glomeruli, sections were incubated with FITC-labeled goat anti-mouse IgG antibody for 30 min at RT. For the detection of complement deposition, kidney sections from cGvHD mice were incubated for 30 min at RT in the dark with FITC-labeled anti-C3 mAb (a kind gift of Dr. S. Izui, University of Geneva) diluted 1:100 in FACS buffer. Bound FITC-labeled mAb was detected by using a fluorescent microscope.

### Flow Cytometry

For analysis by flow cytometry, lymphoid organs were removed and single cell suspensions were prepared by gently passing the organs through a 40 µm nylon mesh into SF-IMDM containing 2% FCS and 0.5% Ciproxin. In order to lyse erythrocytes, spleen cell suspensions were treated with ACK buffer for approximately 1 min. Staining was performed in a 96-well round bottom plate in a total volume of 100 µl containing 50 µl cell suspension (1–2 × 10^7^ cells/ml) and 50 µl diluted antibody mix. Cells were incubated for 30 min on ice in the dark. Cells were washed twice in FACS buffer. When biotinylated antibodies were used, a second staining step (20 min, 4°C, in the dark) was performed for the binding of streptavidin-coupled fluorochromes. If applicable, cells were resuspended in FACS buffer containing 5 µg/ml propidium iodide (Sigma-Aldrich, USA) to exclude dead cells. Intracellular stainings were performed according to standard procedures. In brief, subsequent to surface staining, cells were fixed either with PBS containing 2% paraformaldehyde or with fix/perm buffer (eBioscience, USA) followed by the intracellular staining in FACS buffer containing 0.5% saponin (Sigma-Aldrich, USA) or in permeabilization buffer (eBioscience, USA). Intracellular stainings were incubated for 30 min at 4°C in the dark followed by washing steps in FACS buffer to remove unbound antibodies. Flow cytometry was performed on a FACSCalibur or LSRFortessa flow cytometer (BD Bioscience, USA) and data was analyzed using FlowJo (Tree Star, USA) software. Donor and host cell populations were distinguished by the expression of H2-K^b^ and H2-K^d^. Representative plots of presented key populations are provided in Figure S1 in Supplementary Material.

### Phorbol-12-Myristate-13-Acetate (PMA)/Ionomycin Stimulation for Detection of IFN-γ

Single cell suspensions were stimulated during 4 h using 1 µg/ml ionomycine (Sigma-Aldrich, USA) and 5 ng/ml PMA (Calbiochem, USA) in the presence of 10 µg/ml brefeldin A (Sigma-Aldrich, USA). Cells were harvested and stained by standard intracellular staining procedures (see above).

### Antibodies

Fluorescein isothiocyanate-, phycoerythrin- (PE), allophycocyanin-, Pacific Blue- (PB), Brilliant Violet- (BV), PE-Cy7-, PerCP-Cy5.5, or biotin-labeled monoclonal antibodies specific for CD4 (GK1.5), CD8α (53–6.7), CD8β (YTS-156.7.7), CD25 (PC61), CD44 (IM7), H2-K^b^ (Y3), H2-K^d^ (19.191), TCRβc (H57–597), CD62L (Mel-14), CXCR5 (L138D7), PD-1 (RMP1-30), IFN-γ (XMG1.2), or FoxP3 (FJK-16s) were purchased from BD Bioscience, eBioscience, or BioLegend, or purified from hybridoma culture supernatant and fluorescently labeled in our laboratory according to standard procedures. Antibodies were titrated and used at the lowest dilution that gave the best separation.

## Results

### Prophylactic Administration of IL-2 Complexes

To investigate the effect of prophylactic administration of IL-2 complexes on the development of SLE-like murine cGvHD, BDF1 mice received i.p. injections of IL-2 complexes (either S4B6/IL-2 or JES6/IL-2) on three consecutive days before disease induction. The cGvHD was induced by i.v. injection of parental (DBA/2) lymphocytes from pooled preparations of splenocytes and LN cells. Disease development was followed by measuring the presence of anti-RBC antibodies in the blood, the titers of ANA in the serum, and the incidence of proteinuria over a period of 12 weeks.

**Figure 1:**
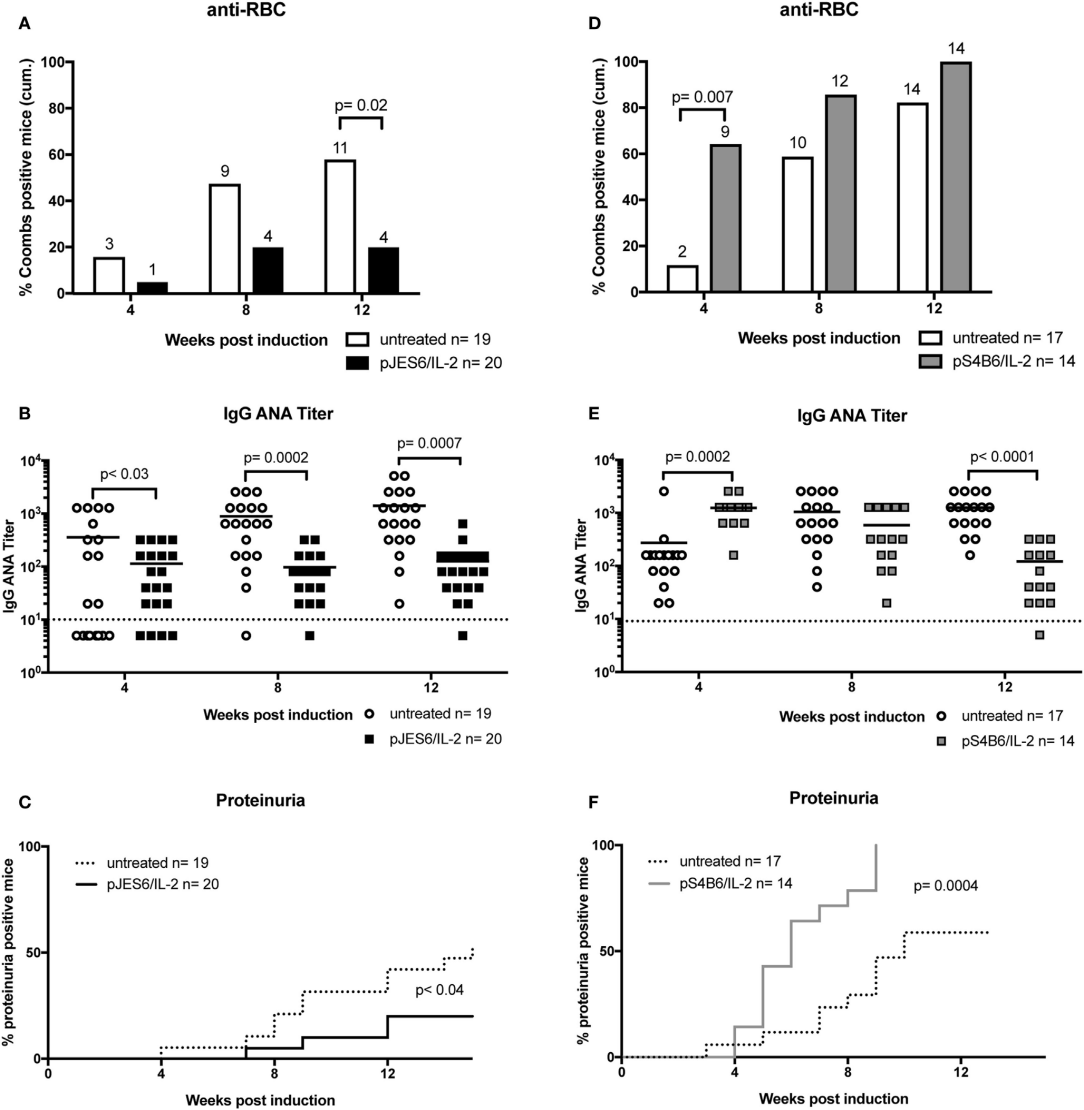
Effect of prophylactic treatment with interleukin-2 (IL-2) complexes on autoimmune symptoms of mice undergoing chronic graft-versus-host-disease (cGvHD). **(A–C)** The beneficial effect of prophylactic JES6/IL-2 treatment on BDF1 mice undergoing cGvHD (pJES6/IL-2: *n* = 20; untreated cGvHD: *n* = 19). **(D–F)** The adverse effect of prophylactic S4B6/IL-2 treatment on BDF1 mice undergoing cGvHD (pS4B6/IL-2: *n* = 14; untreated cGvHD: *n* = 17). **(A,D)** Cumulative frequencies of mice positive for anti-RBC autoantibodies determined at 4, 8, and 12 weeks of cGvHD. Numbers above the bars indicate positive mice. Open bars: untreated cGvHD; Filled bars: cGvHD prophylactically treated with JES6/IL-2 (black) or S4B6/IL-2 (gray) complexes. Statistical significance (*p* < 0.05) was calculated using a two-tailed Fisher’s exact test and is indicated by the *p*-value. **(B,E)** IgG anti-nuclear antibodies (ANA) titer in the serum of cGvHD mice determined 4, 8, and 12 weeks after disease induction. Horizontal bars indicate mean ANA titers in each group. Titers below the dotted line represent mice negative for IgG ANA. Deviations from initially used numbers of mice are indicated at the respective time point. Statistical significance (*p* < 0.05) was calculated using an unpaired Student’s *t*-test and is indicated by the *p*-value. Open circles: untreated cGvHD; filled squares: cGvHD prophylactically treated with JES6/IL-2 (black) or S4B6/IL-2 (gray) complexes. **(C,F)** Frequencies of mice positive for proteinuria as determined by elevated albumin in the urine. Statistical significance (*p* < 0.05) was calculated using the Mantel–Cox test and is indicated by the *p*-value. Dotted line: untreated cGvHD; solid line: cGvHD prophylactically treated with JES6/IL-2 (black) or S4B6/IL-2 (gray) complexes.

**Figure 2:**
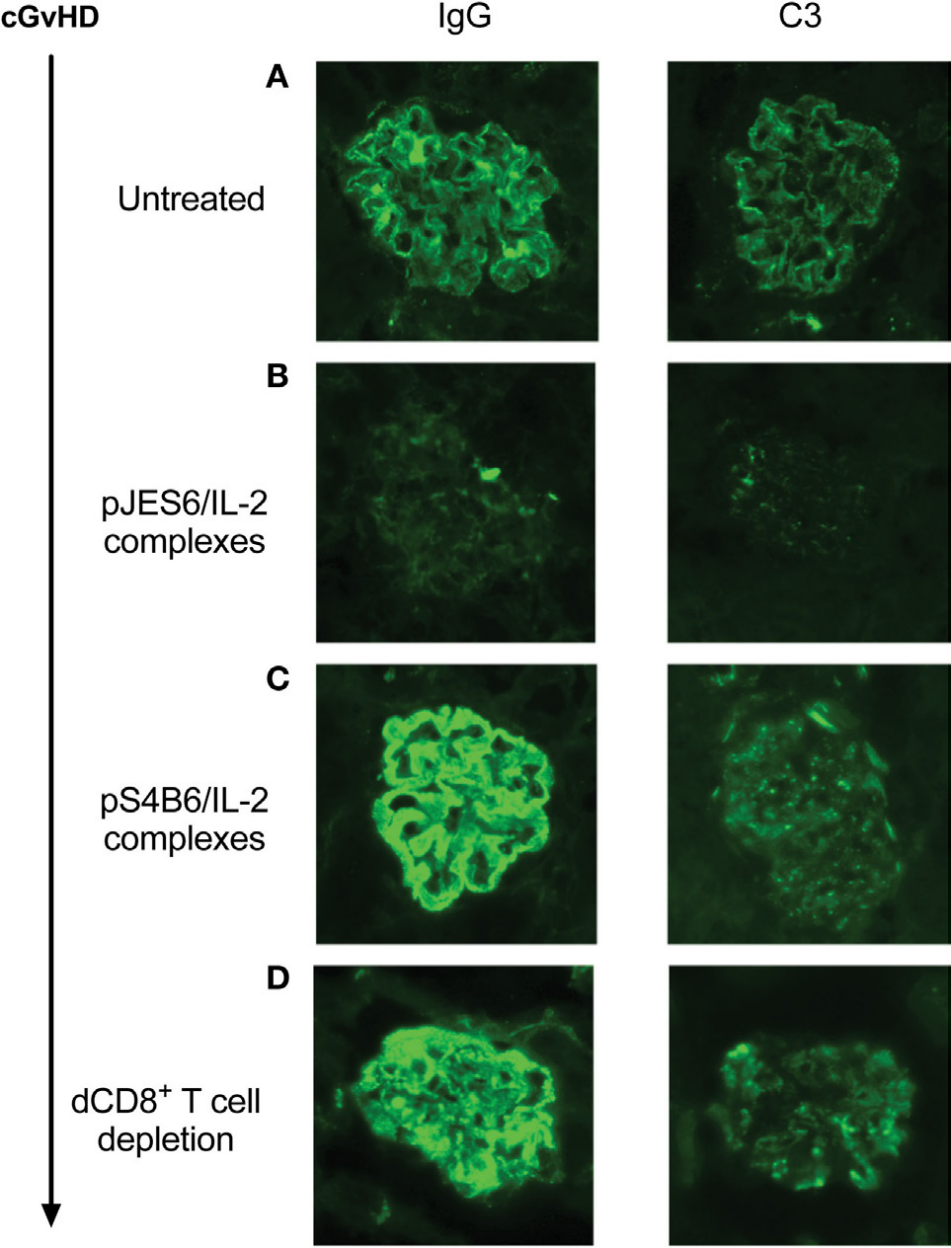
Immunohistological staining of IgG and C3 deposition in glomeruli of proteinuria positive chronic graft-versus-host-disease (cGvHD) mice. Kidneys were obtained from mice positive for proteinuria for <1 week. Cryo sections were prepared and stained for IgG deposition (left panel) or C3 deposition (right panel). Stained sections were analyzed by using a Zeiss Axioscope mounted with a Nikon digital camera DXM 1200F in combination with imaging software (Nikon ACT-1). Photos show representative stainings of kidneys from different experiments. **(A)** Untreated cGvHD mice induced with DBA/2 lymphocytes. **(B)** cGvHD mice as in **(A)**, but prophylactically treated with JES6/interleukin-2 (IL-2). **(C)** cGvHD mice as in **(A)**, but prophylactically treated with S4B6/IL-2. **(D)** Untreated cGvHD mice whose disease was induced with DBA/2 lymphocytes depleted of CD8^+^ T cells.

Prophylactic treatment with JES6/IL-2 complexes (pJES6/IL-2) was highly efficient in ameliorating the SLE-like symptoms of murine cGvHD throughout the observation period. As shown in Figure 1, prophylactic JES6/IL-2 treatment results in reduced frequency of anti-RBC positive mice in the Coombs test as well as decreased titers of ANA in the serum (Figures 1A,B). This was evident at all measured time points. Mice receiving a prophylactic JES6/IL-2 treatment showed delayed onset and reduced incidence of proteinuria (Figure 1C). Analysis of kidneys of proteinuria positive mice by immunohistochemistry showed reduced deposition of immune complexes and complement in those mice that were pretreated with JES6/IL-2 complexes (Figure 2B) compared to untreated cGvHD mice (Figure 2A).

Surprisingly, prophylactic treatment with the S4B6/IL-2 complexes (pS4B6/IL-2) induced stronger autoimmune symptoms and a more aggressive course of disease. At all measured time points, a higher fraction of mice was positive for the production of anti-RBC antibodies in the group receiving prophylactic S4B6/IL-2 treatment (Figure 1D). These mice also had significantly elevated ANA titers in the serum at 4 weeks after disease induction compared to the controls (Figure 1E). The decreased ANA titers measured at 12 weeks after disease induction might be a consequence of the faster kinetics and higher incidence of ICGN in that group. Pretreatment with S4B6/IL-2 complexes resulted in full penetrance of proteinuria by 9 weeks after disease induction (Figure 1F). Moreover, mice pretreated with S4B6/IL-2 complexes showed increased renal deposition of immune complexes and complement (Figure 2C) compared to mice with untreated cGvHD. Besides the significant amounts of albumin secreted by the urine of mice undergoing cGvHD, there is likely a considerable loss of immune globulins that might explain the decreased ANA titers at 12 weeks after disease induction. Despite the reduced number of experimental animals receiving prophylactic S4B6/IL-2 treatment, a statistically significant effect on disease symptoms could be observed that clearly contrasts to the observations in the control group. Although not compared directly within one experiment, the effects of prophylactic S4B6/IL-2 or JES6/IL-2 treatment were in marked contrast to each other and do not arise from variations in the control groups.

In summary, prophylactic treatment with JES6/IL-2 complexes leads to an amelioration of SLE-like symptoms, whereas the prophylactic treatment with S4B6/IL-2 markedly aggravates disease symptoms when compared to mice with the untreated form of murine cGvHD.

### Cellular Mechanism Underlying the Opposing Effects of JES6/IL-2 Versus S4B6/IL-2 Prophylactic Treatments

**Figure 3:**
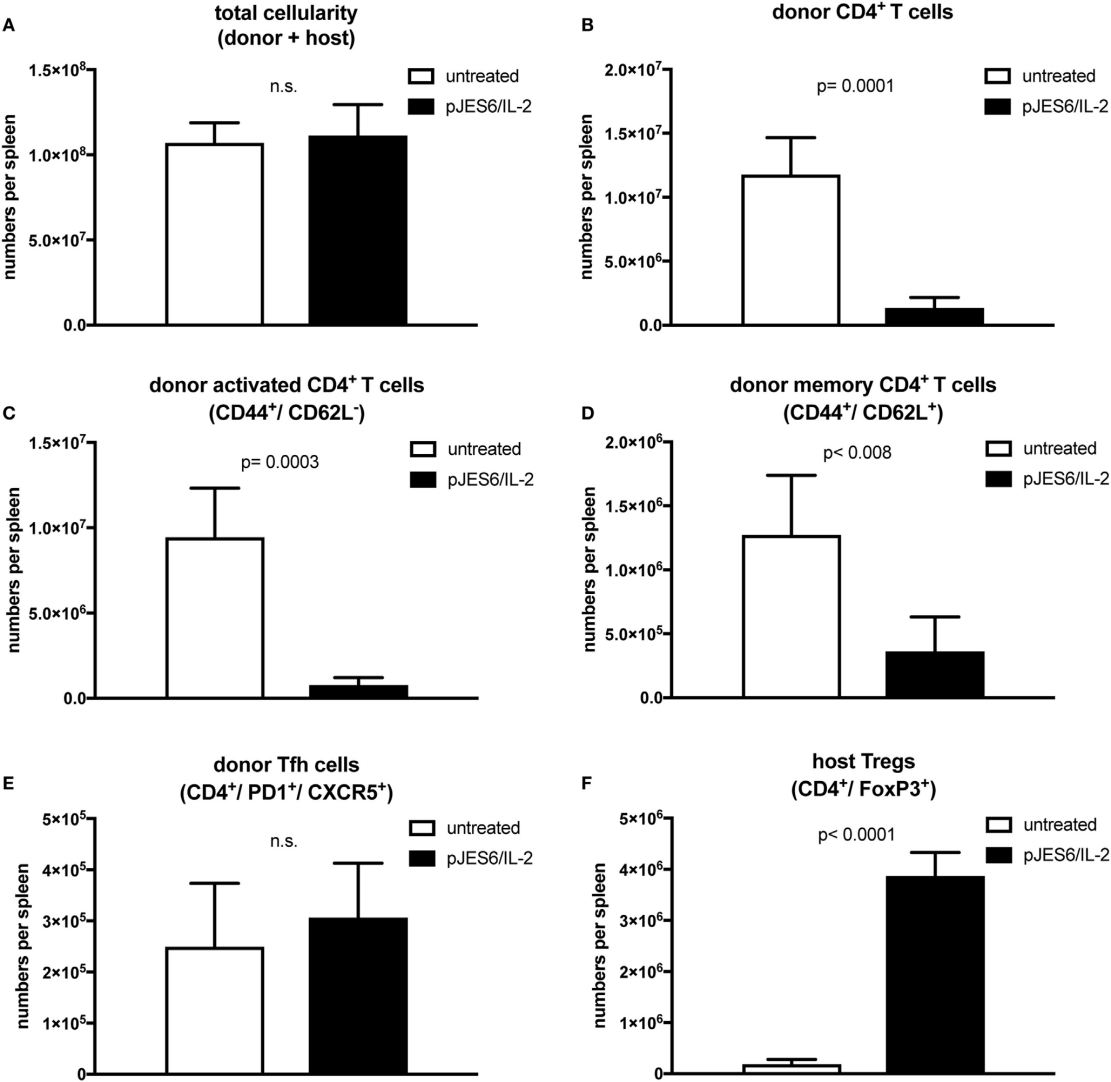
T cell populations in splenocytes from chronic graft-versus-host-disease (cGvHD) mice prophylactically treated with JES6/interleukin-2 (IL-2) complexes*. c*GvHD mice were analyzed in parallel 2 weeks after disease induction. Mean number of total cellularity **(A)** as well as different donor **(B–E)** and host **(F)** T cell subsets per spleen are shown. **(A)** Mean number of total splenocytes (both donor and host origin). **(B)** Mean number of engrafted donor CD4^+^ T cells. **(C)** Mean number of activated donor CD4^+^ T cells expressing CD44. **(D)** Mean number of donor CD4^+^ T cells with central memory phenotype co-expressing CD44 and CD62L. **(E)** Mean number of donor CD4^+^ T cells with follicular helper T cell phenotype co-expressing CXCR5 and PD1. **(F)** Mean number of host regulatory T cells expressing CD4 and FoxP3. White bars: untreated cGvHD (*n* = 4); Black bars: cGvHD prophylactically treated with JES6/IL-2 (*n* = 5). Statistical significance (*p* < 0.05) was calculated using an unpaired Student’s *t*-test and is indicated by the *p*-value. Data are represented as mean values ± SD.

**Figure 4:**
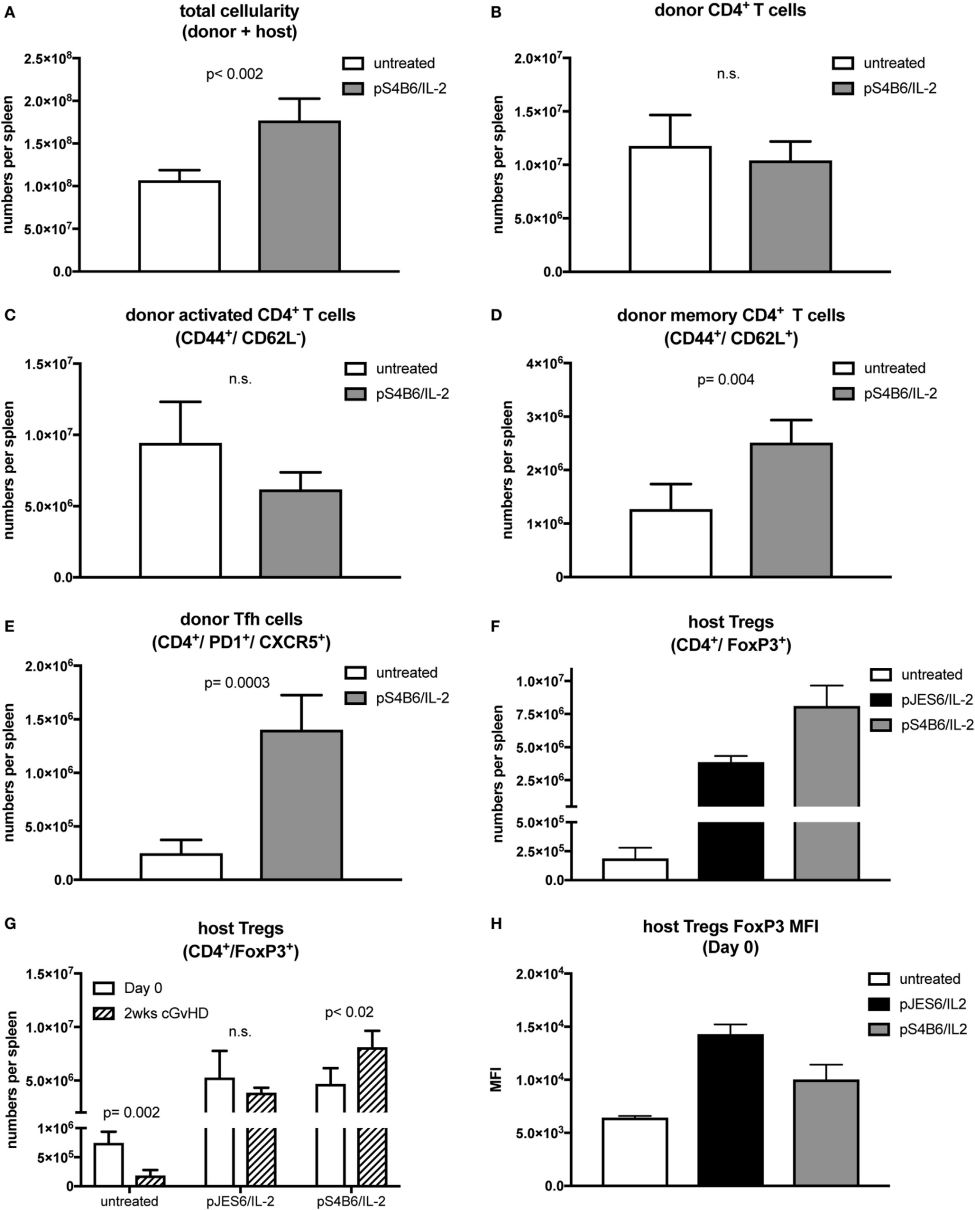
T cell populations in splenocytes from chronic graft-versus-host-disease (cGvHD) mice prophylactically treated with S4B6/interleukin-2 (IL-2) complexes. Mean number of total cellularity **(A)** as well as different donor **(B–E)** and host **(F,G)** T cell subsets per spleen are shown. **(A)** Mean number of total splenocytes (donor and host origin). **(B)** Mean number of engrafted donor CD4^+^ T cells. **(C)** Mean number of activated donor CD4^+^ T cells expressing CD44. **(D)** Mean number of donor CD4^+^ T cells with central memory phenotype co-expressing CD44 and CD62L. **(E)** Mean number of donor CD4^+^ T cells with follicular helper T cell phenotype co-expressing CXCR5 and PD1. **(F)** Mean number of host regulatory T cells (Tregs) expressing CD4 and FoxP3. White bars: untreated cGvHD (*n* = 4); gray bars: cGvHD prophylactically treated with S4B6/IL-2 (*n* = 5); black bars: cGvHD mice prophylactically treated with JES6/IL-2 (*n* = 5). **(G)** Mean numbers of host Tregs in mice left untreated or pretreated with JES6/IL-2 or S4B6/IL-2 at day 0 (just before cGvHD induction) and after 2 weeks of cGvHD. White bars: day 0 (*n* = 4); black patterned bars: 2 weeks cGvHD (*n* = 4–5). **(H)** FoxP3 median fluorescence intensity in host Tregs in mice left untreated or pretreated for 3 days with JES6/IL-2 or S4B6/IL-2 complexes. Statistical significance (*p* < 0.05) was calculated using an unpaired Student’s *t*-test and is indicated by the *p*-value. Data are represented as mean values ± SD.

To gain further insight into how prophylactic administration of IL-2 complexes modifies cellular responses during cGvHD, we performed multicolor flow cytometry analysis on mice, prophylactically treated with IL-2 complexes, 2 weeks after disease induction. Total spleen size was similar to untreated mice (Figure 3A), but numbers of engrafted donor CD4^+^ T cells recovered from the spleens of mice prophylactically treated with JES6/IL-2 complexes was significantly reduced compared to those of the controls (Figure 3B). Moreover, donor CD4^+^ T cells were less activated (Figure 3C) and the population of donor CD4^+^ T cells with a central memory phenotype (CD44^+^/CD62L^+^) was significantly reduced (Figure 3D). Interestingly, there was no significant difference in Tfh cells, identified by staining for the surface markers, PD1 and CXCR5, in the prophylactically JES6/IL-2-treated group compared to controls (Figure 3E). Host Treg compartment expressing the lineage-specific transcription factor FoxP3 was significantly expanded compared to untreated mice (Figure 3F), and absolute numbers of host Tregs were sustained during the first 2 weeks of cGvHD (Figure 4G). The reduced engraftment/expansion as well as decreased activation of donor CD4^+^ T cells might be a consequence of the expanded host Treg compartment (40-fold compared to control group). The finding that prophylactic JES6/IL-2 treatment induced marked increase in FoxP3 expression in host Tregs further supports this assumption (Figure 4H).

Mice treated prophylactically with the S4B6/IL-2 complexes had significantly increased total cellularity of their spleens compared to cGvHD mice in the control group (Figure 4A). In contrast to mice treated prophylactically with JES6/IL-2 complexes, the numbers of total engrafted donor CD4^+^ T cells (Figure 4B) and numbers of activated donor CD4^+^ T cells in cGvHD mice treated prophylactically with S4B6/IL-2 complexes were similar to those found in untreated mice (Figure 4C). On the other hand, central memory donor CD4^+^ T cells were significantly expanded in mice pretreated with S4B6/IL-2 complexes (Figure 4D). Moreover, in these mice, the population of donor CD4^+^ Tfh cells was increased more than fourfold (Figure 4E). Unexpectedly, the extent of host Treg expansion in response to prophylactic treatment with S4B6/IL-2 complexes was even greater compared to the expansion induced by prophylactic JES6/IL-2 treatment (Figure 4F). However, the expansion of host Tregs upon prophylactic S4B6/IL-2 treatment did not ameliorate autoimmune symptoms in contrast to Tregs expanded by prophylactic treatment with JES6/IL-2 complexes. Although we also observed increased FoxP3 expression in S4B6/IL-2 expanded host Tregs (Figure 4H) these cells are unable to efficiently control the induced disease. In these mice elevated numbers of activated donor cells of the CD4^+^ central memory and Tfh subsets might account for the augmented production of autoantibodies and more severe cGvHD observed.

### Therapeutic Administration of IL-2 Complexes

Next, we investigated the influence of the IL-2 complexes on SLE-like symptoms when administrated during ongoing disease. Therefore, we injected either JES6/IL-2 or S4B6/IL-2 complexes therapeutically on three consecutive days starting 4 weeks following the induction of cGvHD by transfer of parental DBA/2 lymphocytes.

**Figure 5:**
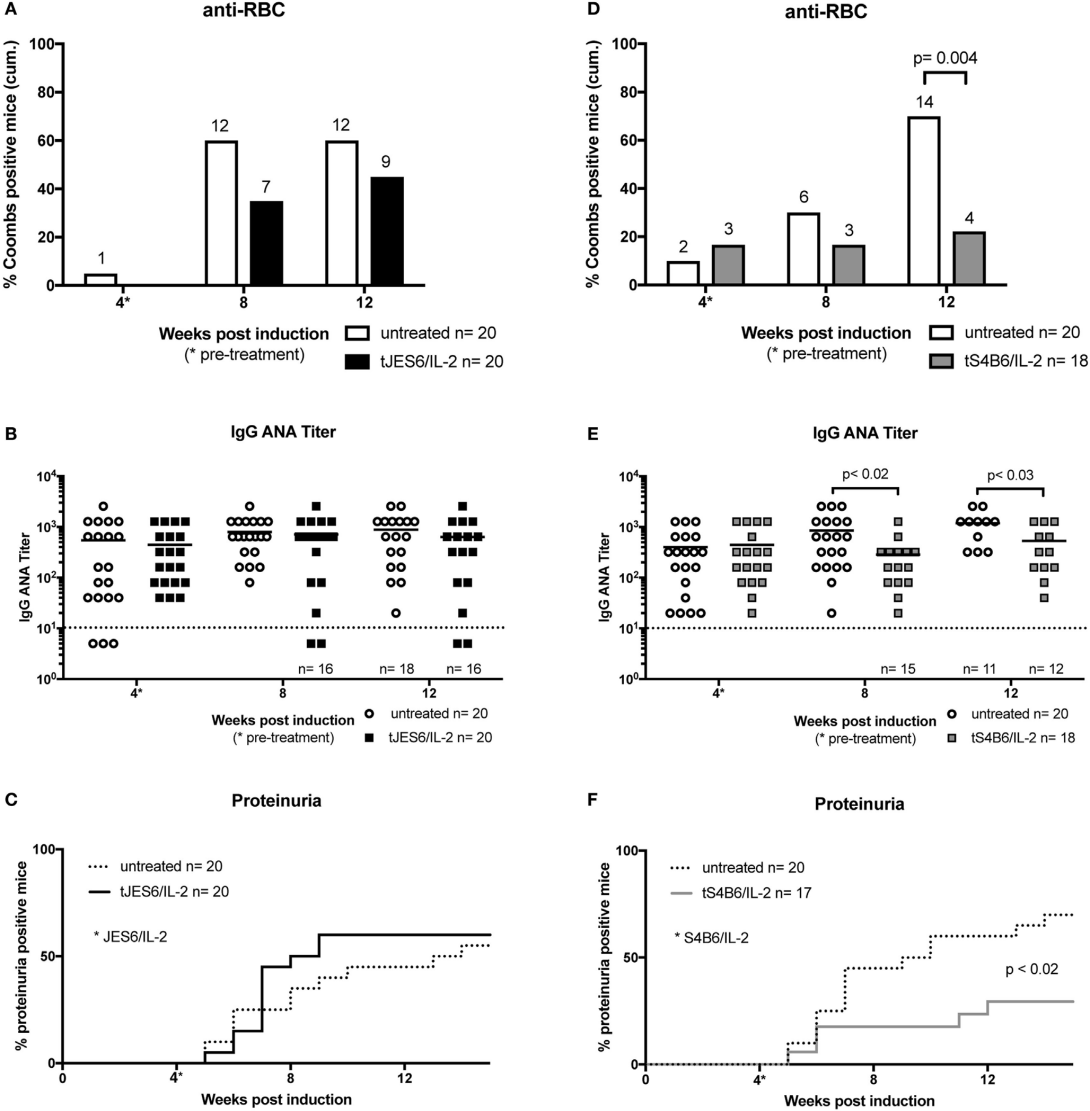
Effect of therapeutic treatment with interleukin-2 (IL-2) complexes on autoimmune symptoms of mice undergoing chronic graft-versus-host-disease (cGvHD). **(A–C)** The efficiency of therapeutic JES6/IL-2 treatment of cGvHD is confined to the production of anti-RBC (tJES6/IL-2: *n* = 20; untreated cGvHD: *n* = 19). **(D–F)** The beneficial effects of therapeutic S4B6/IL-2 treatment of cGvHD (tS4B6/IL-2: *n* = 18; untreated cGvHD: *n* = 20). **(A,D)** Cumulative frequencies of mice positive for anti-RBC autoantibodies determined at 4, 8, and 12 weeks of cGvHD. Numbers above the bars indicate positive mice. White bars: untreated cGvHD; filled bars: cGvHD therapeutically treated with JES6/IL-2 (black) or S4B6/IL-2 (gray) complexes. Statistical significance (*p* < 0.05) was calculated using a two-tailed Fisher’s exact test and is indicated by the *p*-value. **(B,E)** IgG anti-nuclear antibodies (ANA) titer in the serum of cGvHD mice determined 4, 8, and 12 weeks after disease induction. Horizontal bars indicate mean ANA titers in each group. Titers below the dotted line represent mice negative for IgG ANA. Deviations from initially used numbers of mice are indicated at the respective time point. Statistical significance (*p* < 0.05) was calculated using an unpaired Student’s *t*-test and is indicated by the *p*-value. Open circles: untreated cGvHD; filled squares: cGvHD therapeutically treated with JES6/IL-2 (black) or S4B6/IL-2 (gray) complexes. **(C,F)** Frequencies of mice positive for proteinuria as determined by elevated albumin in the urine. Statistical significance (*p* < 0.05) was calculated using the Mantel–Cox test and is indicated by the *p*-value. Dotted line: untreated cGvHD; solid line: cGvHD prophylactically treated with JES6/IL-2 (black) or S4B6/IL-2 (gray) complexes.

The therapeutic treatment with JES6/IL-2 complexes (tJES6/IL-2) had no significant effect on the development of the monitored autoimmune symptoms. Apart from a mild effect on the frequency of anti-RBC positive mice (Figure 5A) ANA titers and frequencies of mice developing proteinuria were comparable in both groups (Figures 5B,C).

In contrast, S4B6/IL-2 complexes, when administrated therapeutically, showed an ameliorating effect on autoimmune symptoms in mice undergoing cGvHD. Following injection of S4B6/IL-2 complexes, the frequency of mice producing anti-RBC antibodies remained constant, whereas the frequency of anti-RBC-producing mice in the control group increased over time (Figure 5D). Moreover, ANA titers measured at time points after the initiation of S4B6/IL-2 therapy were significantly lower compared to the control group that showed a steady increase of ANA titers over time (Figure 5E). The incidence of proteinuria was significantly reduced following S4B6/IL-2 therapy (Figure 5F), most likely as a result of decreased autoantibody production.

Taken together, the data indicate that therapeutic administration of JES6/IL-2 complexes has no significant effect on the symptoms of lupus-like murine cGvHD. This is in contrast to therapeutic administration of S4B6/IL-2 complexes that induce an amelioration of disease symptoms. These results also contrast with the effects of prophylactic administration of these two IL-2 complexes. JES6/IL-2 complexes are effective in prophylactic, but not therapeutic treatment of cGvHD. The reverse is true for S4B6/IL-2 complexes.

### Donor CD8^+^ T Cells Modulate the Pathogenesis of Murine cGvHD

**Figure 6:**
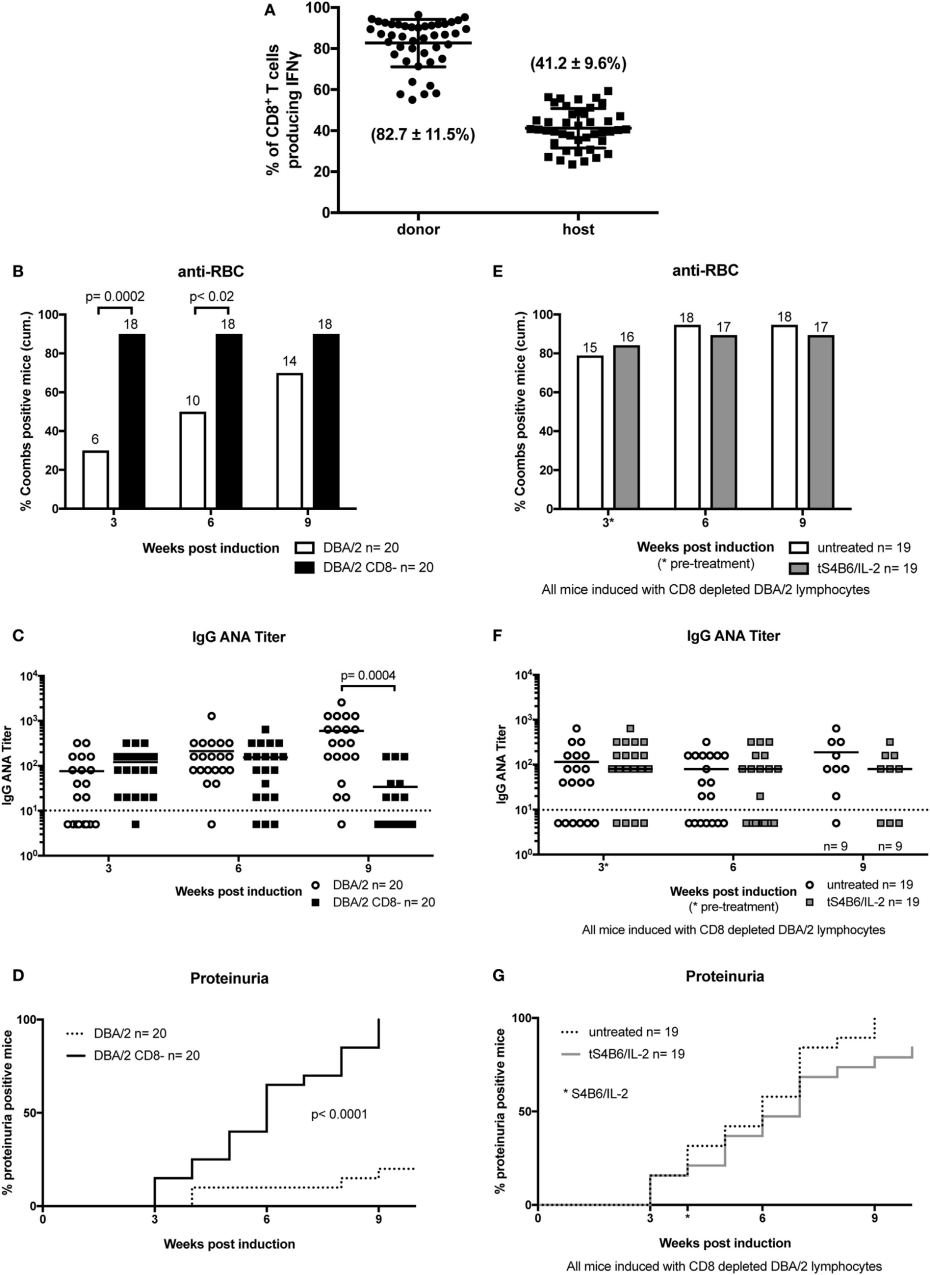
Donor CD8^+^ T cells are important modulators of chronic graft-versus-host-disease (cGvHD) and mediate the beneficial effect of therapeutic S4B6/interleukin-2 (IL-2) therapy. **(A)** Frequencies of IFN-γ producing CD8^+^ T cells of donor (circles) and host (squares) origin in splenocytes of untreated cGvHD mice analyzed at various time points between 2 and 12 weeks after disease induction. **(B–D)** A more severe cGvHD develops when induced with DBA/2 lymphocytes depleted of CD8^+^ T cells (DBA/2 CD8^−^) (DBA/2: *n* = 20; DBA/2 CD8^−^: *n* = 20). **(E–G)** No effect on cGvHD severity with S4B6/IL-2 therapy in the absence of donor CD8^+^ T cells (untreated cGvHD: *n* = 19; tS4B6/IL-2: *n* = 19). **(B,E)** Cumulative frequencies of mice positive for anti-RBC autoantibodies determined at 3, 6, and 9 weeks of cGvHD. Numbers above the bars indicate positive mice. Statistical significance (*p* < 0.05) was calculated using a two-tailed Fisher’s exact test and is indicated by the *p*-value. **(B)** White bars: cGvHD mice induced with DBA/2 lymphocytes; black bars: cGvHD induced with DBA/2 lymphocytes depleted of CD8^+^ T cells. **(E)** White bars: cGvHD induced with DBA/2 lymphocytes depleted of CD8^+^ T cells. **(C,F)** IgG anti-nuclear antibodies (ANA) titer in the serum of cGvHD mice determined 3, 6, and 9 weeks after disease induction. Horizontal bars indicate mean of ANA titers in each group. Titers below the dotted line represent mice negative for IgG ANA. Deviations from initially used number of mice are indicated at the respective time point. Statistical significance (*p* < 0.05) was calculated using an unpaired Student’s *t*-test and is indicated by the *p*-value. **(C)** Open circles: cGvHD induced with DBA/2 lymphocytes; filled squares: cGvHD induced with DBA/2 lymphocytes depleted of CD8^+^ T cells. **(F)** Open circles: untreated cGvHD mice induced with DBA/2 lymphocytes depleted of CD8^+^ T cells; filled squares: cGvHD mice induced with DBA/2 lymphocytes depleted of CD8^+^ T cells and therapeutically treated with S4B6/IL-2 complexes. **(D,G)** Frequencies of mice positive for proteinuria as determined by elevated albumin in the urine. Statistical significance (*p* < 0.05) was calculated using the Mantel–Cox test and is indicated by the *p*-value. **(D)** Dotted line: cGvHD induced with DBA/2 lymphocytes; solid line: cGvHD induced with DBA/2 lymphocytes depleted of CD8^+^ T cells. **(G)** Dotted line: cGvHD induced with DBA/2 lymphocytes depleted of CD8^+^ T cells; solid line: cGvHD induced with DBA/2 lymphocytes depleted of CD8^+^ T cells and therapeutically treated with S4B6/IL-2 complexes.

CD8^+^ T cells are suggested to play an important role in the development of SLE and murine cGvHD (39). The potent capacity of S4B6/IL-2 complexes to expand CD8^+^ T cells (31) and the observation of the beneficial clinical effect of therapeutic S4B6/IL-2 treatment prompted us to further examine the CD8^+^ T cell compartment in mice undergoing cGvHD. Analysis of IFN-γ production in untreated cGvHD mice showed that CD8^+^ T cells of both, donor and host origin, were highly activated irrespective of the time point of the analysis after disease induction. On average, 80% of donor CD8^+^ T cells produced IFN-γ after *in vitro* stimulation, whereas the frequency of IFN-γ-producing cells in the host population was about 40% (Figure 6A). These findings further support the contribution of CD8^+^ T cells during the development of cGvHD. In order to confirm a potential involvement of CD8^+^ T cells and to test the particular contribution of donor and host T cells in the pathogenesis of cGvHD, we followed the development of SLE-like symptoms in the absence of donor CD8^+^ T cells. For this, CD8^+^ T cells were depleted from donor DBA/2 mice by i.v. injection of 200 µg YTS-156, a monoclonal anti-CD8β antibody, 4 days prior to harvesting donor cells for cGvHD induction. The high efficiency of *in vivo* donor CD8^+^ T cell depletion was confirmed by flow cytometry analysis of the donor lymphocyte preparation (data not shown).

The results show that depletion of donor CD8^+^ T cells results in a much more severe cGvHD. Almost all mice induced with CD8^+^ T cell-depleted donor inoculum were positive for the presence of anti-RBC antibodies already after 3 weeks of cGvHD, whereas in the control group, receiving non-depleted donor cells, the frequency of anti-RBC positive mice reached 80% with delayed kinetics (Figure 6B). ANA titers in the two groups were not significantly different during the first 6 weeks of cGvHD. However, at later time points, ANA titers of mice induced with donor CD8^+^ T cell depleted cells decrease, whereas the titers of the other group continued to increase (Figure 6C). Again, decreased ANA titers at 12 weeks of cGvHD might be due to the greater incidence of proteinuria in this group, showing earlier onset and full penetrance by 9 weeks after disease induction. Immunohistological analysis of kidneys of cGvHD mice induced with donor inoculum depleted of CD8^+^ T cells revealed a marked increase of immune complex and complement deposition (Figure 2D) in contrast to untreated cGvHD mice induced with non-depleted donor cells (Figure 2A).

### Beneficial Effect of Therapeutic S4B6/IL-2 Treatment Depends on Donor CD8^+^ T Cells

The importance of the donor CD8^+^ T cell compartment in determining the severity of disease symptoms raised the following questions: (i) To what extent can the observed effect of S4B6/IL-2 complexes be attributed to donor or host CD8^+^ T cells and (ii) whether the effect is reproducible in the absence of donor CD8^+^ T cells? To test this, cGvHD was induced by transfer of donor lymphocytes from DBA/2 mice previously injected with the CD8β-depleting mAb YTS-156. After 4 weeks of ongoing disease, one group of mice was treated with S4B6/IL-2 complexes on three consecutive days. No significant improvement of the disease symptoms was observed upon therapeutic S4B6/IL-2 treatment in cGvHD mice injected with CD8^+^ T cell-depleted donor lymphocytes. We failed to observe any significant difference, in any measured parameter, throughout the experiment between the two experimental groups (Figures 6E–G). These results provide strong evidence for the idea that donor CD8^+^ T cells are required for S4B6/IL-2 complexes to ameliorate SLE-like symptoms in mice undergoing cGvHD.

## Discussion

Murine chronic GvHD resulting from the injection of DBA/2 lymphocytes into BDF1 mice leads to autoimmune symptoms closely resembling SLE in man. Although the beneficial effect of IL-2 in autoimmunity has been established some time ago (29), severe side effects impeded its application in the clinics. The discovery that such adverse reactions could be prevented when IL-2 was administrated as immune complex bound to anti-IL-2 mAb opened up new therapeutic approaches for the treatment of autoimmune diseases like SLE. Herein, we investigated the prophylactic and therapeutic effect of two IL-2 complexes (JES6/IL-2 and S4B6/IL-2) on SLE-like symptoms resulting from chronic GvHD.

Prophylactic administration of JES6/IL-2 complexes ameliorated cGvHD symptoms and protected BDF1 mice to a large extent from developing fatal ICGN. In these treated mice, fewer donor CD4^+^ T cells had engrafted in the spleen 2 weeks after transfer. Donor CD4^+^ T cells are central in the pathogenesis of cGvHD by providing help to host B cells; this leads to the production of disease driving autoantibodies (40). Hence, amelioration of disease upon JES6/IL-2 pretreatment is a likely consequence of the reduced engraftment and/or functioning of donor CD4^+^ T cells. Moreover, donor CD4^+^ T cells in JES6/IL-2-pretreated mice were phenotypically less activated and generated reduced numbers of central memory T cells. This may results from enhanced suppressive capacity of the host Treg compartment following the prophylactic treatment with JES6/IL-2 complexes. It is well established that Tregs can regulate effector T cell responses by suppressing their activation and differentiation into effector subsets (41). In this regard, it is conceivable that prophylactic treatment with JES6/IL-2 complexes expands a host Treg population capable of suppressing alloreactive donor CD4^+^ T cells.

In marked contrast, prophylactic treatment with S4B6/IL-2 complexes significantly enhanced SLE-like symptoms and induced a more severe course of the disease. Mice pretreated with S4B6/IL-2 complexes had increased splenomegaly as well as significantly higher numbers of donor CD4^+^ T cells with a central memory phenotype. Memory T cells have lower activation thresholds and respond to secondary antigenic stimulation with augmented effector function (42), thereby possibly enhancing autoimmunity. Moreover, only pretreatment with S4B6/IL-2 complexes induced differentiation of donor CD4^+^ Tfh cells expressing PD1 and CXCR5. Expression of CXCR5 facilitates the localization of Tfh cells to CXCL13 producing B cells and the initiation of germinal center. As Tfh cells contribute to survival, affinity maturation, isotype class-switching, and differentiation of germinal center B cells (9), it is likely that exacerbated SLE-like symptoms in S4B6/IL-2-pretreated mice resulted from enhanced germinal center reactions leading to augmented production of pathogenic autoantibodies. In support of this hypothesis, a pathogenic role of Tfh cells has been recently established in other murine lupus models and in human SLE (43).

Whether administration of S4B6/IL-2 complexes directly promotes differentiation of donor CD4^+^ T cells into Tfh cells is unclear. First, the biological half-life of IL-2 complexes is relatively short (<4 h) (31), thereby arguing against a direct contribution on donor CD4^+^ T cell differentiation, since donor cells were transferred approximately 24 h after the last injection of IL-2 complexes. Second, S4B6/IL-2 complexes failed to induce expression of Tfh cell-specific markers in normal mice not undergoing cGvHD (data not shown). For these reasons, we expect the effect of S4B6/IL-2 pretreatment on donor Tfh differentiation is indirect and likely involves cells of the host. Moreover, consistent with previous reports (37), we found that pretreatment with S4B6/IL-2 complexes leads to marked expansion of dendritic cells expressing CD11c and MHC-II (data not shown). Whether the expanded dendritic cell compartment contributes to exacerbated cGvHD in S4B6/IL-2 pretreated mice, perhaps by providing enhanced co-stimulation factors, requires further investigation.

Another surprising finding was the effect of S4B6/IL-2 pretreatment on the host Treg compartment. As previously reported (31) and in agreement with our own data, at the time of cGvHD induction, host Treg compartments were expanded equally, efficiently upon prophylactic treatment with JES6/IL-2 or S4B6/IL-2 complexes. Nevertheless, mice pretreated with S4B6/IL-2 complexes developed exacerbated cGvHD symptoms, whereas JES6/IL-2 pretreated mice were largely protected from disease. Although mice pretreated with S4B6/IL-2 or JES6/IL-2 complexes have not been compared directly in one experiment, the observed effects on the course of the disease induced by either complex are strikingly different and it is highly unlikely that these differences are artifacts resulting from variations in the control groups. Surprisingly, the host Treg compartment in the first 2 weeks of cGvHD in S4B6/IL-2 pretreated mice markedly expanded, but was unable to control the disease. These rather contradictory findings suggest functional differences in the capacity of Tregs stimulated either by S4B6/IL-2 or JES6/IL-2 complexes. Since mAb S4B6 blocks the epitope necessary for the interaction of IL-2 with CD25, S4B6/IL-2 complexes largely prevent signaling *via* high-affinity IL-2 receptors, but allow for signal transduction through low-affinity IL-2 receptors. This stimulation through low-affinity IL-2 receptor might induce Treg survival and expansion, but may not maintain suppressive functionality. Interestingly, Tregs from CD25^−/−^ Bim^−/−^ mice, where IL-2-dependent survival and function have been uncoupled, were shown to be less suppressive *in vitro* and unable to prevent autoimmunity *in vivo* (44). Thus, maintenance of Treg functionality in our model seems to be critically dependent on IL-2 signals transduced by high-affinity IL-2 receptors, which apparently cannot be substituted by IL-2 signaling *via* the low-affinity receptor. Therefore, it is likely that Tregs expanded upon pretreatment with S4B6/IL-2 complexes are poorly functional and unable to control alloreactive donor CD4^+^ T cells compared to those expanded by prophylactic JES6/IL-2 treatment. Notably, the ratio of host Tregs to donor CD4^+^ T cells in S4B6/IL-2 complex-pretreated mice was decreased compared to the ratio in mice pretreated with JES6/IL-2 complexes. Since these ratios were similar in both groups at the initiation of cGvHD (day 0), these differences must have accumulated during the first 2 weeks of cGvHD (compare Figures 3B and 4B,F). These differences may be a result of differences in suppressive capacity in host Tregs in the two different groups at the time of donor cell transfer.

Therapeutic administration of JES6/IL-2 complexes following the induction of cGvHD, on the other hand, showed no significant effect on cGvHD symptoms and the general course of the disease. Our findings demonstrate a critical timing for JES6/IL-2 treatment in the cGvHD model of lupus. Since pathogenic mechanisms are already well established at the time of JES6/IL-2 therapy (4 weeks after cGvHD induction), Treg expansion was not able to ameliorate the ongoing autoimmunity. In this regard, it has to be considered that, in an ongoing disease, JES6/IL-2 complexes may stimulate not only Treg, but also engrafted alloreactive donor CD4^+^ T cells that have been previously activated and express high-affinity IL-2 receptors. However, while therapeutic administration of JES6/IL-2 complexes stimulated donor CD4^+^ T cells expressing the high-affinity IL-2 receptor, this did not result in aggravated disease symptoms. It is conceivable that a parallel increase of the suppressive capacity in the Treg compartment might counteract the stimulatory effect on donor CD4^+^ T cells and thereby prevented disease exacerbation. Notably, therapeutic administration of JES6/IL-2 complexes in a spontaneous model of lupus was shown to ameliorate ICGN (45), arguing in favor for our hypothesis that stimulation of donor CD4^+^ T cells by JES6/IL-2 complexes canceled out the beneficial effect of Treg stimulation. Moreover, in contrast to cGvHD, spontaneous lupus is a slowly developing disease that is not driven by a high number of alloreactive donor cell populations and, therefore, might be more susceptible to therapeutic intervention with JES6/IL-2 complexes. Another explanation for the inefficiency of therapeutic JES6/IL-2 treatment to control the disease might be linked to the fact that during the course of cGvHD, the numbers of host Tregs significantly decrease as shown for untreated cGvHD mice at 2 weeks. Thus, fewer host Tregs are present to respond to JES6/IL-2 complexes resulting in a smaller expanded regulatory compartment with poor suppressive capacity.

Finally, therapeutic administration of S4B6/IL-2 complexes significantly ameliorated SLE-like symptoms in ongoing cGvHD. Our findings strongly suggest that this improvement is dependent on donor CD8^+^ T cells, since mice injected with CD8^+^ T cell-depleted donor cells were not affected by therapeutic S4B6/IL-2 treatment. It might well be envisaged that S4B6/IL-2 complexes stimulate the activation and differentiation of host-reactive donor CD8^+^ T cells into functional cytotoxic T lymphocytes (CTLs), thereby inducing a mild form of acute GvHD. B cells are known to be one of the first host lymphocyte population targeted by alloreactive CTLs in acute GvHD (46). Thus, therapeutic S4B6/IL-2 might have ameliorated cGvHD symptoms by enhancing a donor CD8^+^ T cell-mediated alloresponse against autoreactive host B cells leading to reduced production of autoantibodies. In support of our hypothesis it was recently shown that IL-21 could also induce the stimulation of such anti-host responses of donor CD8^+^ T cells resulting in amelioration of cGvHD symptoms (47). The fact that a more severe cGvHD results from the injection of CD8^+^ T cell-depleted donor cells further supports a regulatory role of this population in murine cGvHD.

In conclusion, JES6/IL-2 complexes efficiently ameliorated cGvHD in our model only when administrated prophylactically, possibly by acting during priming and initiation of the disease. Due to improved diagnosis of SLE and a considerable lag time until clinical manifestation of severe lupus, JES6/IL-2 complexes might be considered as potential approach to prevent more severe symptoms in those patients, where SLE is recognized early enough. In contrast, S4B6/IL-2 aggravated SLE-like symptoms when administrated prophylactically and ameliorated disease only when given therapeutically. With prophylactic treatment, we propose an indirect effect of S4B6/IL-2 complexes on donor CD4^+^ T cell differentiation leading to more severe disease symptoms. With therapeutic treatment of an ongoing cGvHD, the ameliorating effect was likely due to direct effects of S4B6/IL-2 complexes on donor CD8^+^ T cells inducing their activation and subsequent enhancement of alloresponses directed against host lymphocytes, e.g., host B cells. Results obtained from therapeutic treatment with S4B6/IL-2 complexes may not be directly relevant for the treatment of SLE. While SLE patients exhibit an increased cytotoxicity among their CD8^+^ T cells, this has been correlated with increased disease activity. Increased cytotoxic activity of (autoimmune) CD8^+^ T cells would release self-antigens from target cells resulting in further stimulation of autoreactive lymphocytes. Nevertheless, the therapeutic administration of S4B6/IL-2 complexes and experiments using CD8^+^ T cell-depleted donor cells point to a potential regulatory role of donor CD8^+^ T cells in the pathogenesis of murine cGvHD. In summary, the administration of IL-2/mAb complexes has marked effects on the course of cGvHD in this murine model. Whether our findings can be adapted to a treatment of autoimmune diseases in humans is an area for further investigation.

## Ethics Statement

Animal experiments were carried out within institutional guidelines (authorization number 1888 and 2434 from Cantonal Veterinarian Office, Basel).

## Author Contributions

AR conceived the study and designed experiments. All authors performed experiments and analyzed the data. AR and SH wrote the paper.

## Conflict of Interest Statement

The authors declare no financial or commercial conflicts of interest. The handling Editor declared a past co-authorship with one of the authors (AR).

## Supplementary Material

Figure S1 Representative FACS plots of key populations shown in Figures 3 and 4. Top row indicates the ancestor gating of plots shown below. Squares within plots indicate population gates. Numbers adjacent to population gates indicate frequency of gated population in the ancestor gates. First row: untreated chronic graft-versus-host-disease mice. Second row: prophylactically JES6/IL-2 treated mice. Third row: prophylactically S4B6/IL-2 treated mice.

## References

[1] Tsokos GC Mechanisms of disease systemic lupus erythematosus. N Engl J Med (2011) 365(22):2110–21. 10.1056/NEJMra1100359 22129255

[2] van der Vlag J Berden JH. Lupus nephritis: role of antinucleosome autoantibodies. Semin Nephrol (2011) 31(4):376–89. 10.1016/j.semnephrol.2011.06.009 21839371

[3] Relle M Schwarting A Role of MHC-linked susceptibility genes in the pathogenesis of human and murine lupus. Clin Dev Immunol (2012) 2012:1–15. 10.1155/2012/584374 PMC3385965 22761632

[4] Tsao BP. An update on genetic studies of systemic lupus erythematosus. Curr Rheumatol Rep (2002) 4(4):359–67. 10.1007/s11926-002-0046-5 12126589

[5] Foster MH T cells and B cells in lupus nephritis. Semin Nephrol (2007) 27(1):47–58. 10.1016/j.semnephrol.2006.09.007 17336688 PMC1997284

[6] Shlomchik MJ Craft JE Mamula MJ. From T to B and back again: positive feedback in systemic autoimmune disease. Nat Rev Immunol (2001) 1(2):147–53. 10.1038/35100573 11905822

[7] Schroeder K Herrmann M Winkler TH. The role of somatic hypermutation in the generation of pathogenic antibodies in SLE. Autoimmunity (2013) 46(2):121–7. 10.3109/08916934.2012.748751 23181829

[8] Faderl M Klein F Wirz OF Heiler S Albertí-Servera L Engdahl C Two distinct pathways in mice generate antinuclear antigen-reactive B cell repertoires. Front Immunol (2018):9. 10.3389/fimmu.2018.00016 29403498 PMC5786517

[9] Crotty S Follicular helper CD4 T cells (T-FH). Annu Rev Immunol (2011) 29:621–63. 10.1146/annurev-immunol-031210-101400 21314428

[10] Sawaf M Dumortier H Monneaux F. Follicular helper T cells in systemic lupus erythematosus: why should they be considered as interesting therapeutic targets? J Immunol Res (2016) 2016:1–13. 10.1155/2016/5767106 27635407 PMC5011227

[11] Yildirim-Toruner C Diamond B. Current and novel therapeutics in the treatment of systemic lupus erythematosus. J Allergy ClinImmunol (2011) 127(2):303–12. 10.1016/j.jaci.2010.12.1087 21281862 PMC3053574

[12] Navarra SV Guzmán RM Gallacher AE Hall S Levy RA Jimenez RE Efficacy and safety of belimumab in patients with active systemic lupus erythematosus: a randomised, placebo-controlled, phase 3 trial. Lancet (2011) 377(9767):721–31. 10.1016/S0140-6736(10)61354-2 21296403

[13] Jacobi AM Huang WQ Wang T Freimuth W Sanz I Furie R Effect of long-term belimumab treatment on B cells in systemic lupus erythematosus. Arthritis Rheum (2010) 62(1):201–10. 10.1002/art.27189 20039404 PMC2857977

[14] Crampton SP Morawski PA Bolland S. Linking susceptibility genes and pathogenesis mechanisms using mouse models of systemic lupus erythematosus. Dis Model Mech (2014) 7(9):1033–46. 10.1242/dmm.016451 25147296 PMC4142724

[15] Andrews BS Eisenberg RA Theofilopoulos AN Izui S Wilson CB McConahey PJ Spontaneous murine lupus-like syndromes. Clinical and immunopathological manifestations in several strains. J Exp Med (1978) 148(5):1198–215. 10.1084/jem.148.5.1198 309911 PMC2185049

[16] Perry D Sang A Yin Y Zheng YY Morel L. Murine models of systemic lupus erythematosus. J Biomed Biotechnol (2011) 2011:1–19. 10.1155/2011/271694 21403825 PMC3042628

[17] Eisenberg R The chronic graft-versus-host model of systemic autoimmunity. Curr Dir Autoimmun (2003) 6:228–44. 10.1159/000066864 12408055

[18] Gleichmann E Vanelven EH Vanderveen JPW A systemic lupus-erythematosus (SLE)-like disease in mice induced by abnormal T-B-cell cooperation – preferential formation of autoantibodies characteristic of SLE. Eur J Immunol (1982) 12(2):152–9. 10.1002/eji.1830120210 6978818

[19] Van der Veen F Rolink AG Gleichmann E. Diseases caused by reactions of T lymphocytes to incompatible structures of the major histocompatibility complex. IV. Autoantibodies to nuclear antigens. Clin Exp Immunol (1981) 46(3):589–96. 6978221 PMC1536298

[20] Rolink AG Gleichmann H Gleichmann E Diseases caused by reactions of T lymphocytes to incompatible structures of the major histocompatibility complex. VII. Immune-complex glomerulonephritis. J Immunol (1983) 130(1):209–15. 6183348

[21] Malek TR. The biology of interleukin-2. Annu Rev Immunol (2008) 26:453–79. 10.1146/annurev.immunol.26.021607.090357 18062768

[22] Létourneau S Krieg C Pantaleo G Boyman O. IL-2-and CD25-dependent immunoregulatory mechanisms in the homeostasis of T-cell subsets. J Allergy Clin Immunol (2009) 123(4):758–62. 10.1016/j.jaci.2009.02.011 19348914

[23] Alcocervarela J Alarconsegovia D Decreased production of and response to interleukin-2 by cultured lymphocytes from patients with systemic lupus-erythematosus. J Clin Invest (1982) 69(6):1388–92. 10.1172/JCI110579 6979554 PMC370212

[24] Lieberman LA Tsokos GC. The IL-2 defect in systemic lupus erythematosus disease has an expansive effect on host immunity. J Biomed Biotechnol (2010) 2010:1–6. 10.1155/2010/740619 20625413 PMC2896881

[25] Gillis S Smith KA Long term culture of tumour-specific cytotoxic T cells. Nature (1977) 268(5616):154–6. 10.1038/268154a0 145543

[26] Rosenberg SA Mule JJ Spiess PJ Reichert CM Schwarz SL Regression of established pulmonary metastases and subcutaneous tumor mediated by the systemic administration of high-dose recombinant interleukin-2. J Exp Med (1985) 161(5):1169–88. 10.1084/jem.161.5.1169 3886826 PMC2187617

[27] Rosenstein M Ettinghausen SE Rosenberg SA Extravasation of intravascular fluid mediated by the systemic administration of recombinant interleukin-2. J Immunol (1986) 137(5):1735–42. 3528289

[28] Fyfe GA Fisher RI Rosenberg SA Sznol M Parkinson DR Louie AC Long-term response data for 255 patients with metastatic renal cell carcinoma treated with high-dose recombinant interleukin-2 therapy. J Clin Oncol (1996) 14(8):2410–1. 10.1200/JCO.1996.14.8.2410 8708739

[29] Gutierrez-Ramos JC Andreu JL Revilla Y Viñuela E Martinez C. Recovery from autoimmunity of MRL/lpr mice after infection with an interleukin-2/vaccinia recombinant virus. Nature (1990) 346(6281):271–4. 10.1038/346271a0 1973822

[30] Boyman O Surh CD Sprent J. Potential use of IL-2/anti-IL-2 antibody immune complexes for the treatment of cancer and autoimmune disease. Expert Opin Biol Ther (2006) 6(12):1323–31. 10.1517/14712598.6.12.1323 17223740

[31] Boyman O Kovar M Rubinstein MP Surh CD Sprent J. Selective stimulation of T cell subsets with antibody-cytokine immune complexes. Science (2006) 311(5769):1924–7. 10.1126/science.1122927 16484453

[32] Spangler JB Tomala J Luca VC Jude KM Dong S Ring AM Antibodies to interleukin-2 elicit selective T cell subset potentiation through distinct conformational mechanisms. Immunity (2015) 42(5):815–25. 10.1016/j.immuni.2015.04.015 25992858 PMC4439582

[33] Létourneau S Leeuwen EM Krieg C Martin C Pantaleo G Sprent J IL-2/anti-IL-2 antibody complexes show strong biological activity by avoiding interaction with IL-2 receptor alpha subunit CD25. Proc Natl Acad Sci U S A (2010) 107(5):2171–6. 10.1073/pnas.0909384107 20133862 PMC2836659

[34] Boyman O Krieg C Letourneau S Webster K Surh CD Sprent J. Selectively expanding subsets of T cells in mice by injection of interleukin-2/antibody complexes: implications for transplantation tolerance. Transplant Proc (2012) 44(4):1032–4. 10.1016/j.transproceed.2012.01.093 22564618

[35] Lee SY Cho ML Oh HJ Ryu JG Park MJ Jhun JY Interleukin-2/anti-interleukin-2 monoclonal antibody immune complex suppresses collagen-induced arthritis in mice by fortifying interleukin-2/STAT5 signalling pathways. Immunology (2012) 137(4):305–16. 10.1111/imm.12008 23167249 PMC3530086

[36] Webster KE Walters S Kohler RE Mrkvan T Boyman O Surh CD In vivo expansion of Treg cells with IL-2-mAb complexes: induction of resistance to EAE and long-term acceptance of islet allografts without immunosuppression. J Exp Med (2009) 206(4):751–60. 10.1084/jem.20082824 19332874 PMC2715127

[37] Jin GH Hirano T Murakami M. Combination treatment with IL-2 and anti-IL-2 mAbs reduces tumor metastasis via NK cell activation. Int Immunol (2008) 20(6):783–9. 10.1093/intimm/dxn036 18448458

[38] Krieg C Létourneau S Pantaleo G Boyman O. Improved IL-2 immunotherapy by selective stimulation of IL-2 receptors on lymphocytes and endothelial cells. Proc Natl Acad Sci U S A (2010) 107(26):11906–11. 10.1073/pnas.1002569107 20547866 PMC2900642

[39] Puliaeva I Puliaev R Via CS. Therapeutic potential of CD8+ cytotoxic T lymphocytes in SLE. Autoimmun Rev (2009) 8(3):219–23. 10.1016/j.autrev.2008.07.045 18725326 PMC3215296

[40] Rolink AG Gleichmann E. Allosuppressor- and allohelper-T cells in acute and chronic graft-vs-host (GVH) disease. III. Different Lyt subsets of donor T cells induce different pathological syndromes. J Exp Med (1983) 158(2):546–58. 10.1084/jem.158.2.546 6224882 PMC2187357

[41] Itoh M Takahashi T Sakaguchi N Kuniyasu Y Shimizu J Otsuka F Thymus and autoimmunity: production of CD25(+)CD4(+) naturally anergic and suppressive T cells as a key function of the thymus in maintaining immunologic self-tolerance. J Immunol (1999) 162(9):5317–26. 10228007

[42] Sallusto F Geginat J Lanzavecchia A. Central memory and effector memory T cell subsets: function, generation, and maintenance. Annu Rev Immunol (2004) 22:745–63. 10.1146/annurev.immunol.22.012703.104702 15032595

[43] Blanco P Ueno H Schmitt N. T follicular helper (Tfh) cells in lupus: activation and involvement in SLE pathogenesis. Eur J Immunol (2016) 46(2):281–90. 10.1002/eji.201545760 26614103

[44] Barron L Dooms H Hoyer KK Kuswanto W Hofmann J O’Gorman WE Cutting edge: mechanisms of IL-2-dependent maintenance of functional regulatory T cells. J Immunol (2010) 185(11):6426–30. 10.4049/jimmunol.0903940 21037099 PMC3059533

[45] Yan JJ Lee JG Jang JY Koo TY Ahn C Yang J IL-2/anti-IL-2 complexes ameliorate lupus nephritis by expansion of CD4(+)CD25(+)Foxp3(+) regulatory T cells. Kidney Int (2017) 91(3):603–15. 10.1016/j.kint.2016.09.022 27914701

[46] Puliaev R Puliaeva I Welniak LA Ryan AE Haas M Murphy WJ CTL-promoting effects of CD40 stimulation outweigh B cell-stimulatory effects resulting in B cell elimination and disease improvement in a murine model of lupus. J Immunol (2008) 181(1):47–61. 10.4049/jimmunol.181.1.47 18566369 PMC2613003

[47] Nguyen V Rus H Chen C Rus V CTL-promoting effects of IL-21 counteract murine lupus in the parent -> F1 graft-versus-host disease model. J Immunol (2016) 196(4):1529–40. 10.4049/jimmunol.1501824 26792801

