# Supplementary figures and images for: Prophylactic and Therapeutic Effects of Interleukin-2 (IL-2)/Anti-IL-2 Complexes in Systemic Lupus Erythematosus-Like Chronic Graft-Versus-Host Disease
Source: Front Immunol. 2018 Apr 4;9:656. doi: 10.3389/fimmu.2018.00656 (PMC5893767; doi:10.3389/fimmu.2018.00656)

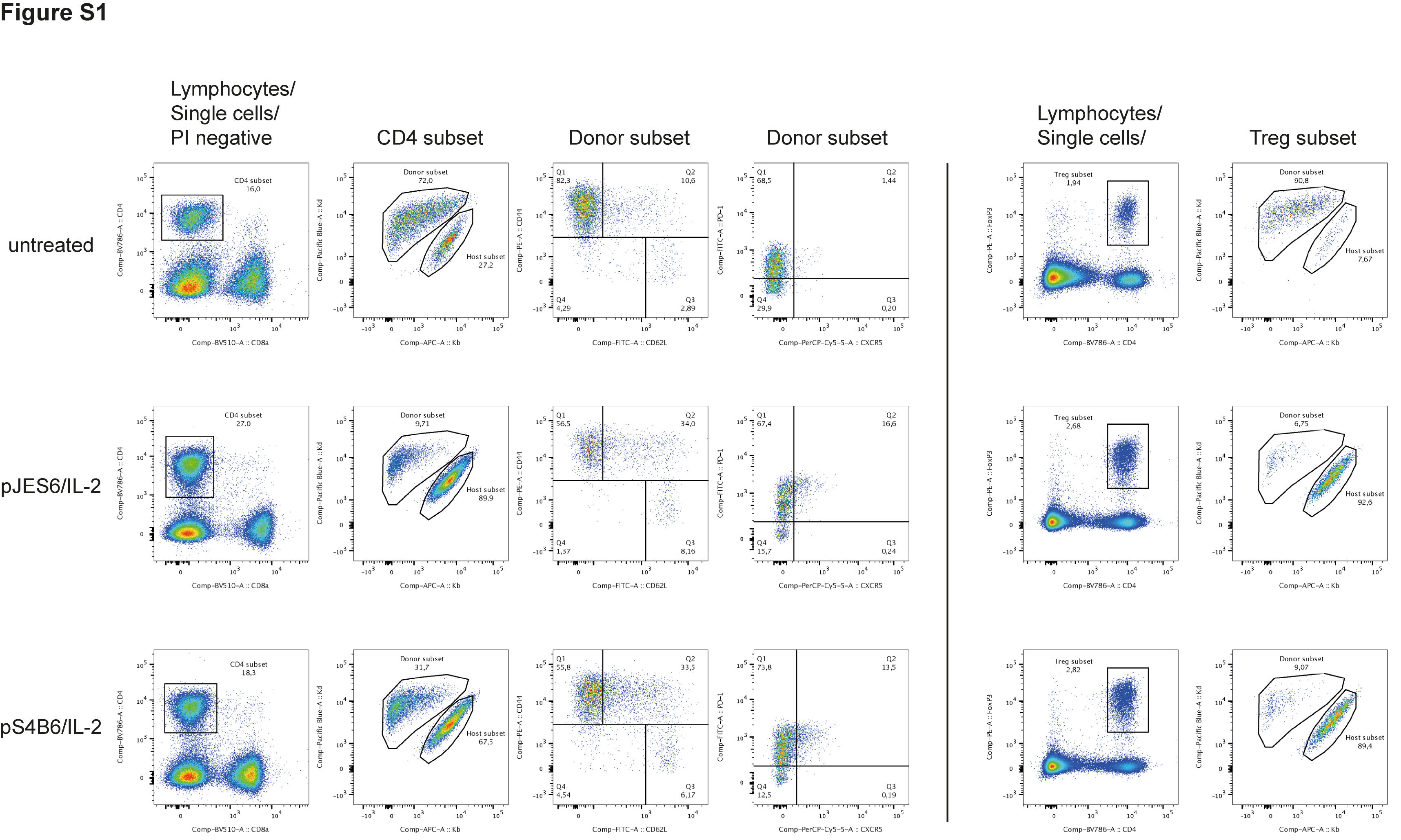

Supplement: Figure S1 — Representative FACS plots of key populations shown in Figures 3 and 4. Top row indicates the ancestor gating of plots shown below. Squares within plots indicate population gates. Numbers adjacent to population gates indicate frequency of gated population in the ancestor gates. First row: untreated chronic graft-versus-host-disease mice. Second row: prophylactically JES6/IL-2 treated mice. Third row: prophylactically S4B6/IL-2 treated mice. [file image_1.tif]
